# Activation of the aryl hydrocarbon receptor in mature adipocytes does not impact diet-induced obesity in mice

**DOI:** 10.12688/wellcomeopenres.25201.1

**Published:** 2026-02-16

**Authors:** Julia Vlachaki Walker, Benjamin Wiggins, Alice Burke, Marian Dore, Alessandro Sardini, Chris Schiering

**Affiliations:** 1 Faculty of Medicine, Imperial College London Institute of Clinical Sciences, London, England, UK; 2 MRC London Institute of Medical Sciences, London, England, UK; 3 King's College London Wolfson Centre for Age-Related Diseases, London, England, UK; 4 Immunoregulation Laboratory, The Francis Crick Institute, London, England, UK

**Keywords:** Obesity, metabolic syndrome, adipose tissue, adipocytes, adipocyte precursors, AHR

## Abstract

**Background:**

Despite rising awareness and advances in the field, the prevalence of obesity continues to increase. Current weight-loss treatments are often ineffective long-term, and some are associated with serious side effects. For this reason, alternative pathways need to be explored in order to identify novel pharmacological targets for the treatment of obesity. In this study, we aim to investigate the role of the ligand-activated transcription factor, aryl hydrocarbon receptor (AHR), in the development of obesity and metabolic syndrome.

**Methods & Results:**

Using global and adipocyte-specific *Ahr* knock-out mouse models, we show that even though global *Ahr* deletion has some protective effects from diet-induced obesity and metabolic syndrome, adipocyte-specific deletion of *Ahr* has no effect. Furthermore, using a variety of techniques to isolate mature adipocytes, such as flotation, *in-vitro* differentiation, and translating ribosome affinity purification, we show no evidence that *Ahr* is expressed in mature white adipocytes. Finally, using single-cell RNA sequencing on cells from a *Cyp1a1* fate reporter mouse line, in which eYFP can be used as a readout of AHR activation, we show that the AHR pathway is active in adipose stem and precursor cells. Using bulk RNA sequencing on adipocyte precursors stimulated with an AHR ligand *in-vitro*, we show evidence suggesting that *Ahr* may be involved in the control of adipocyte differentiation.

**Conclusions:**

Our study indicates that *Ahr* may play an important role in adipocyte precursors, particularly in the initiation of adipocyte differentiation. We suggest that further investigation into this mechanism may be important to fully elucidate AHR’s effects in obesity.

## Plain Language Summary

Despite advances in the field, obesity continues to affect millions of people’s health and quality of life. Often, weight-loss treatments are not effective long-term and some can have serious side effects. Because of this, scientists are looking for ways to further understand and treat obesity. In this study, the researchers look a protein called aryl hydrocarbon receptor (AHR) to see if it plays a role in obesity and related health problems.

The researchers in this study show that removing AHR entirely in the mouse (by genetic modification) can affect the how the mice become obese and associated metabolic problems. As fat cells are the main driving force of obesity, the researchers also remove AHR only in fat cells. However this time, the researchers show no effect on weight-gain and metabolism. They also showed that, unlike what was previously believed, AHR is not found in fat cells, even though it can be found in fat tissue.

Finally, the researchers show that within fat tissue, AHR is mostly found in cells that fat cells originate from (precursor cells), and suggest that AHR may play an important role in early fat cell development. This could be an important clue in finding new ways to treat or prevent obesity, and more research is needed to understand this process better.

## List of symbols and abbreviations

3-MC            3-Methylcholanthrene

AHR             aryl hydrocarbon receptor

AHRE          aryl hydrocarbon response element

APC             adipocyte precursor cell

ASPC           adipose stem and precursor cell

BAT             brown adipose tissue

ChIP            Chromatin Immunoprecipitation

CLAMS      Comprehensive Lab Animal Monitoring System

DIO             Diet-induced obesity

FACS          fluoresce-activated cell sorting

FICZ          6-formylindolo[3,2-b]carbazole

GTT           glucose tolerance test

gWAT         perigonadal white adipose tissue

HFD          High-fat diet

I3C            Indole-3-carbinol

ITT            insulin tolerance test

iWAT         inguinal white adipose tissue

LFD           Low-fat diet 

## Introduction

Despite increased awareness of its effects on health and quality of life, obesity remains a global epidemic. According to the World Health organisation, 1 in 8 people world-wide were living with obesity in 2022. Even though certain types of monogenic obesity have been identified, these make up a small proportion of obesity cases ( Loos & Yeo, 2022). Genome-wide association studies (GWAS) have also identified genetic variants, which are believed to work in conjunction with environmental factors to increase the risk of developing obesity ( Frayling *et al*., 2007; van der Klaauw & Farooqi, 2015). For this reason, we chose to focus on diet-induced obesity (DIO) in this study.

Obesity itself can have detrimental effects on a person’s quality of life. However, what makes this condition even more a concern is its association with comorbidities, such as type 2 diabetes mellitus (T2DM), cancer, and cardiovascular disease ( DeFronzo *et al*., 2015; Font-Burgada *et al*., 2016; Kivimaki *et al*., 2022). Obesity is characterised by the expansion of adipose tissue, accompanied by immune cell infiltration and inflammation, as well as ectopic lipid accumulation and lipotoxicity in peripheral organs ( Jager *et al*., 2007; Li *et al*., 2004; Rotter *et al*., 2003; Yu *et al*., 2002). These are believed to contribute to the development of insulin resistance which, in turn, results in reduced glucose uptake and hyperglycaemia, driving further insulin release, which over time can lead to the development of T2DM ( Czech, 2017; DeFronzo *et al*., 2015). Together, these symptoms of chronic low-grade inflammation, insulin resistance, raised fasting blood glucose, and elevated blood pressure are referred to as metabolic syndrome. A variety of approaches have been taken to treat obesity, however, most reported limited success and can pose other health risks. Even following successful weight loss interventions, weight re-gain is often observed ( Look, 2014; van Baak & Mariman, 2019). It is therefore crucial to continue researching new targets for the treatment of obesity and metabolic syndrome.

One receptor that has received attention as a potential therapeutic target in the treatment of obesity and metabolic syndrome is the aryl hydrocarbon receptor (AHR). AHR is a ligand-activated transcription factor originally characterised for its involvement in 2,3,7,8-Tetrachlorodibenzodioxin (TCDD) toxicity ( Mandal, 2005; Poland *et al*., 1971). The halogenated hydrocarbons initially studied, usually a byproduct of industrial processes, are associated with a plethora of pathologies, such as chloracne and wasting syndrome in factory workers exposed to these chemicals ( Poland *et al*., 1971). For this reason, AHR activation was initially believed to be detrimental to health, and a large proportion of studies focused on the deleterious effects of its activation by these xenobiotic ligands. Subsequent research has identified, naturally occurring AHR ligands derived from sources including cruciferous vegetables, tryptophan, or produced by the gut microbiota. One of the key differences between naturally occurring and xenobiotic ligands is their ability to be metabolised by the body. Naturally occurring ligands result in transient AHR activation ( Chen *et al*., 1995; Wei *et al*., 1998), whilst xenobiotic ligands remain in the body for extended periods of time due to the inability of the body to metabolise them by the same mechanism ( Bohonowych & Denison, 2007).

AHR ligands tend to be lipophilic and can accumulate within the adipose tissue ( Baker *et al*., 2013; Chevrier *et al*., 2000). Pre-clinical studies have shown that supplementing a high-fat diet (HFD) with the AHR ligand DIM or pro-ligand, indole-3-carbinol (I3C), resulted in reduced weight gain and fat pad weight, accompanied by a reduction in plasma triglycerides, glucose and FFA, and well as a reduction in plasma insulin and insulin resistance ( Choi *et al*., 2012; Yang *et al*., 2017). Paradoxically, studies on a global AHR KO mouse model have shown these mice exhibit increased glucose tolerance and insulin sensitivity compared to WT mice ( Wang *et al*., 2011) and are partly protected from developing DIO, metabolic syndrome and adipose tissue immune cell infiltration and inflammation ( Xu *et al*., 2015). Studies looking at AHR deletion in mature adipocytes in mice fed HFD have also found opposing results. One study saw increased weight gain, fat mass and adipocyte size compared to control mice ( Baker *et al*., 2015), while another observed reduced weight gain and smaller adipocyte size, improved glucose clearance and insulin sensitivity and reduced plasma insulin compared to littermate controls ( Huang *et al*., 2022). Together these studies suggest AHR may play a role in the development of DIO and metabolic syndrome, potentially through actions in mature adipocytes, however the mechanisms involved and whether AHR activation is beneficial or detrimental is unclear.

Our study aims to carefully dissect the role of AHR in adipose tissue in relation to obesity and metabolism.

## Methods

### Mice

Mice were bred and maintained in the Imperial College Central Biological Services Unit at the Hammersmith Hospital Campus, in specific pathogen-free conditions (SPF), on a 12-hour light cycle, at ~21 °C and provided with food and water *ad libitum*. Cages were supplemented with nesting material, gnawing sticks, and, unless otherwise specified, fed normal chow diet. Diets were purchased from Research Diets Inc. Low-fat diet (LFD; 10% fat; D12450J) and high-fat diet (HFD; 60% fat; D12492) were purchased, as a purified diet, or purified diet supplemented with 1,000 mg/kg I3C.

Where possible, diet studies were started at 6 weeks of age, and no later than 10, as indicated in the figure legends. Where possible, littermates were used as the control group and treatment groups were split evenly within cages. Where experimental groups were not based on genotype, mice were randomly allocated to experimental groups by the experimenter in an unbiased approach. Where experimental groups were determined by genotype, mice of similar ages were selected from each group if possible. Where possible, both males and females were used for *in vivo* experiments, however, due to lack of availability of mice, only males were used in some studies. The number of mice used for each experiment was limited by the availability of mice of the correct age and genotype. This means that experiments are underpowered to detect subtle effects, and therefore, if genotype would have had small effects on the measurements taken, these effects may have gone undetected. No formal criteria were established a priori for the inclusion or exclusion of animals or data points in this study. One mouse was excluded from these studies due to the development of hydrocephaly. All other animals that underwent experimental procedures were included in the analysis. Experimenters were not blinded, however all measures were quantitative and therefore unlikely to be affected by experimenter bias.

For studies on global AHR deletion, the Bradfield AHR KO mouse was used ( Schmidt *et al*., 1996). For the study of Adipocyte-specific AHR deletion, *AdipoqCre* mice ( Eguchi *et al*., 2011) (Jackson Laboratory 010803) crossed to *AHR ^fl/fl^* were used. The same *AdipoqCre* line was also crossed to *NuTRAP* mice ( Roh *et al*., 2017) to generate mice with GFP expression targeted to adipocyte ribosomes. Finally, *Cyp1a1Cre* mice ( Henderson *et al*., 2015) were crossed to R26 ^LSL-eYFP^ mice ( Srinivas *et al*., 2001) to create a Cyp1a1 reporter line.

Mice were euthanised by cervical dislocation, followed by cessation of circulation to confirm death. All researchers were trained in animal handling and assessed regularly by Biological Services technical staff to ensure competence for all procedures used. Approval for this work was obtained from the local ethics committee (AWERB), Imperial College London (PPL: PCADA4D83). All procedures were carried out in accordance with UK Home Office regulations and Animal Scientific Procedure Act 1986.

### Enzymatic digestion of mouse adipose tissue

Adipose tissue was collected in PBS with 0.5% BSA (fatty-acid-free; Sigma A3803-100G) and kept on ice until ready to process. Tissue was minced using scalpels and digested in DMEM (Thermo Fisher, 41965039) containing 2% BSA, 12.5 mM HEPES, 0.2 mg/ml liberase TL (Sigma, 5401020001) and 1/100 DNase (Sigma, 10104159001) for 30min at 37 ^o^C with constant agitation at 180 rpm. The digestion was stopped by adding one volume DMEM, and the sample centrifuged at 250xg for 10min to separate the stromovascular (SVF) and adipocyte fractions.

For mRNA analysis, both fractions were collected into separate tubes and washed once with PBS. RNA was extracted using the TRIzol™ Plus RNA Purification Kit (Life Technologies, 12183555) and cDNA prepared and real time qPCR (RT-qPCR) performed as described below.

### Flow cytometry & cell sorting of mouse adipose tissue cells

For flow cytometry, adipose tissue was digested and the SVF collected as above. Cells were strained through a 100 μm cell strainer, centrifuged at 300xg for 3min to pellet and red blood cells lysed by resuspending in ACK lysis buffer (0.15 M NH _4_Cl, 1 mM KHCO _3_, 0.1 mM Na _2_EDTA, pH 7.4) for 2min. DMEM was added to stop the lysis, cells strained through a 40 μm cell strainer and centrifuged at 300xg for 3min to pellet. Cells were resuspended in DMEM and counted and an appropriate number of cells aliquoted per condition and panel. Cells were washed in FACS buffer (3% FBS, 2 mM EDTA) and pelleted at 300xg for 3min. Fc receptor blockade was carried out using TruStain Fc Block (1:500; Biolegend, 101320) for 15min on ice. For flow cytometry experiments, cells were incubated in Zombie near infra-red (ZNIR; 1:1000; Biolegend, 423105) on ice for 10min. Cells were incubated in antibody mix for 20min on ice (CD45 PerCP-Cy5.5, 1:200, Biolegend 103132; CD31 AF647, 1:200, Biolegend 102516; Pdgfrα BV605, 1:100, Biolegend 135916). Cells were washed and resuspended in FACS buffer for analysis on the BD LSR II analyser (FACSDiva software, BD). Analysis was performed on FlowJo (FlowJo_v10.8.1, FLOWJO LLC).

For cell sorting ZNIR was omitted. After staining cells were passed through a 40 μm cell strainer and resuspended in FACS buffer containing 10 ng/ml DAPI (Sigma, D9542-1MG). BD FACS Aria III or BD FACS Aria Fusion, running FACSDiva software (BD) was used. For RNA analysis, a 85 μm nozzle was used and sorted cells were collected in RLT lysis buffer from the RNeasy® Plus Micro kit (Qiagen, 74034) supplemented with 1% β- Mercaptoethanol (Sigma, M3148-100ML) and immediately snap-frozen. For in vitro cell culture, a 100 μm nozzle was used and sorted cells were collected in 100% FBS. Cells were then centrifuged at 300 x g for 5min and re-suspended in ITS medium ( Table 1) for counting and plating. Media was replaced every 2 days and cells used once confluent without passaging.

**Table 1.** ITS medium.

Component	Supplier	Catalogue No.	Concentration
DMEM, low glucose	SLS	10-014-CV	60%
MCDB 201	Sigma	M6770	40%
FBS	Sigma	F7524-500ML	2%
Insulin-transferrin-Selenium (ITS)	Life Tech	41400-045	0.02%
L-ascorbic acid-2-2-phosphate	Sigma	A8960-5G	0.1 mM
Fibroblast growth factor (FGF) basic	Bio-Techne	3139-FB	10 ng/ml
Pen/Strep	Sigma	P4333-100ML	0.5%
Gentamicin	Sigma	G1264-50MG	0.2%

### Cytokine array

To measure changes in cytokine release from cultured adipocyte precursors, conditioned media was collected from the treated cells, centrifuged at 300 x g for 5min to remove any debris, the supernatant collected to a fresh tube, and snap-frozen until ready to process. The Mouse XL Cytokine Array Kit (R&D, ARY028) was used as per the manufacturer’s instructions, pooling the conditioned media from 6 repeats for each treatment condition.

### Real time qPCR

For analysis of sorted cells, RNA extraction was performed using the RNeasy ^®^ Plus Micro kit.

For analysis of snap frozen adipose tissue, one lobe per mouse was homogenized in 1ml Trizol using a Precellys homogenizer. For analysis of cultured cells, media was aspirated, and cells washed once with PBS. 500ml Trizol was then added and cells homogenized by pipetting. RNA was extracted using the TRIzol™ Plus RNA Purification Kit. cDNA was prepared using the Applied Biosystems™ High-Capacity cDNA Reverse Transcription Kit, followed by quantitative real-time PCR using SYBR green (Fisher Scientific, A25742). Transcription levels were normalized to *B2m* expression and where ΔΔCt is shown, values were normalized to control. For primers, see Table 2.

**Table 2.** Mouse RT-qPCR primers.

Target	Forward 5’-3’	Reverse 5’-3’
*Adipoq*	GCACTGGCAAGTTCTACTGCAA	GTAGGTGAAGAGAACGGCCTTGT
*Ahr*	GCCCTTCCCGCAAGATGTTAT	TCAGCAGGGGTGGACTTTAAT
*B2m*	TTCTGGTGCTTGTCTCACTGA	CAGTATGTTCGGCTTCCCATTC
*Cyp1a1*	CAATGAGTTTGGGGAGGTTACTG	CCCTTCTCAAATGTCCTGTAGTG
*Emr1*	CTTTGGCTATGGGCTTCCAGTC	GCAAGGAGGACAGAGTTTATCGTG
*Fabp4*	AAGGTGAAGAGCATCATAACCCT	TCACGCCTTTCATAACACATTCC

### Nuclei Isolation & Chromatin Immunoprecipitation (ChIP)

Both lobes of BAT or WAT per mouse were partly-thawed on ice, and immediately chopped using scalpels. The tissue was homogenized in nuclear preparation buffer ( Table 3) using a Dounce homogenizer and filtered through a 100 μm strainer. Nuclei were fixed using 1% formaldehyde for 4 minutes and quenched using glycine. Samples were washed twice in 1% NP-40 and resuspended in nuclear lysis buffer ( Table 3) and counted. 2 x 10 ^6^ nuclei/ml sonicated for 4 cycles (30s on, 30s off) in 150 μl aliquots using the Bioruptor Pico (Diagenode). Chromatin samples were diluted 1:1 in 2x RIPA buffer ( Table 3) and precleared by incubating with protein A/G dynabeads (1:1 mix of A & G dynabeads; Thermo Fisher, 10004D, 10002D) for 1hr. Input sample was taken at this stage, following preclearing. Precleared samples were incubated with 10 μg/ml anti-AHR (Enzo BML-SA210-0100) antibody or the equivalent volume of water overnight (unbound control). Chromatin-antibody complexes or unbound controls were then incubated with A/G dynabeads for 3 hours. Beads were washed twice with each of: low-salt RIPA buffer, high-salt RIPA buffer, LiCl buffer and TE buffer, and eluted using elution buffer ( Table 3). Samples were de-crosslinked overnight and purified using the QIAquick PCR Purification Kit (Qiagen, 28106). AHR recruitment was assessed by running a quantitative real-time PCR using primers for the aryl hydrocarbon response element (AHRE) region on the *Cyp1a1* gene, and a negative control region (-0.8 kb and -3.6 kb from the AHRE respectively). Primers used for ChIP-qPCR: *Cyp1a1* -0.8 kb 5’-3’ AAGCATCACCCTTTGTAGCC 3’-5’ CAGGCAACACAGAGAAGTCG, *Cyp1a1* -3.6 kb 5’-3’ GCTCTTTCTCTGCCAGGTTG3’-5’ GGCTAAGGGTCACAATGGAA.

**Table 3.** Nuclei isolation & ChIP buffers.

Buffer	Component	Supplier	Catalogue No.	Concentration
Nuclear preparation buffer	HEPES, pH 7.5	Sigma	H0887-100ML	10 mM
	KCl	Sigma	P5405-250G	10 mM
	MgCl _2_	Sigma	63069-100ML	1.5 mM
	NP-40 (Igepal)	Sigma	I8896-100ML	0.1%
	Sucrose	Sigma	84097-250G	250 mM
	Dithiothreitol (DTT)	Sigma	D0632-10G	0.2 mM
	Phenylmethylsulfonyl fluoride (PMSF)	Sigma	10837091001	1 mM
Nuclear lysis buffer	Tris, pH 8	Life Tech	AM9856	10 mM
	EDTA	Thermo Fisher	AM9260G	1 mM
	Sodium Dodecyl Sulfate (SDS)	Fisher Scientific	10607633	0.25%
	Triton-X	Sigma	T8787-100ML	1%
	Protease inhibitor	Sigma	P8340-5ML	1x
2x RIPA buffer	Triton-X	Sigma	T8787-100ML	1.1%
	Sodium deoxycholate (DOC)	Sigma	D6750-100G	0.2%
	NaCl	Sigma	S5886-500G	280 mM
	Protease inhibitor	Sigma	P8340-5ML	1x
Low-salt RIPA buffer	SDS	Fisher Scientific	10607633	0.1%
	Triton-X	Sigma	T8787-100ML	1%
	EDTA	Thermo Fisher	AM9260G	1 mM
	Tris-HCl pH 8.1	Life Tech	AM9856	20 mM
	NaCl	Sigma	S5886-500G	140 mM
	DOC	Sigma	D6750-100G	0.1%
High-salt RIPA buffer	SDS	Fisher Scientific	10607633	0.1%
	Triton-X	Sigma	T8787-100ML	1%
	EDTA	Thermo Fisher	AM9260G	1 mM
	Tris-HCl, pH 8.1	Life Tech	AM9856	20 mM
	NaCl	Sigma	S5886-500G	500 mM
	DOC	Sigma	D6750-100G	0.1%
LiCl buffer	LiCl	Sigma	62476-100G-F	250 mM
	NP-40	Sigma	I8896-100ML	0.5%
	DOC	Sigma	D6750-100G	0.5%
	EDTA	Thermo Fisher	AM9260G	1 mM
	Tris-HCl, pH 8.1	Life Tech	AM9856	10 mM
TE buffer	NaHCO3	Sigma	S6297	100 mM
	SDS	Fisher Scientific	10607633	1%

### Translating Ribosome Affinity Purification (TRAP)

BAT or WAT of *Adipoq-Cre NuTRAP* mice was partly thawed on ice and immediately roughly minced using scalpels. The tissue was homogenised in IP buffer ( Table 4) using a Dounce homogeniser. The sample was transferred to an eppendorf and further homogenised by vortexing three times. The sample was centrifuged to remove debris and excess fat and adipocyte ribosomes bound to an anti-GFP antibody (Abcam, ab290). The ribosome-antibody complex was subsequently bound to protein G dynabeads. Following five washes in high-salt buffer ( Table 4), the ribosomes were lysed from the beads and the RNA extracted using the PureLink™ RNA Mini Kit (Thermo Fisher, 12183018A). cDNA was prepared, and RT-qPCR performed as described above.

**Table 4.** TRAP buffers.

Buffer	Component	Supplier	Catalogue No.	Concentration
IP buffer	Tris, pH 7.5	Life Tech	15504020	50 mM
	MgCl _2_	Sigma	63069-100ML	12 mM
	KCl	Sigma	P5405-250G	100 mM
	NP-40	Sigma	I8896-100ML	1%
	Cycloheximide	Sigma	C7698-1G	100 μg/ml
	Heparin	Fisher Scientific	BP2524-100	1 mg/ml
	DTT	Sigma	D0632-10G	2 mM
	RNAsin	Promega	N2515	0.2 U/μl
	Protease inhibitor	Sigma	P8340-5ML	1x
High Salt Wash Buffer	Tris, pH 7.5	Life Tech	15504020	50 mM
	MgCl _2_	Sigma	63069-100ML	12 mM
	KCl	Sigma	P5405-250G	300 mM
	NP-40	Sigma	I8896-100ML	1%
	Cycloheximide	Sigma	C7698-1G	100 μg/ml
	DTT	Sigma	D0632-10G	2 mM

### Single-cell RNA sequencing

For single-cell RNA sequencing, adipose tissue was digested, and cells sorted from SVF as above using the 100 μm nozzle and cells collected in 10% FBS. Libraries were prepared using the 10x Single Cell 3' v3 kit according to manufacturer specifications. CellRanger v4.0.0 was used to map the FASTQ filed to the mouse genome and Seurat 4.1.0 was used for scRNAseq analysis. Cells with a minimum of 100 features (unique genes), less than 12.5% mitochondrial genes, and a range of 500 to 40000 molecules detected were used, excluding any cells not meeting these criteria. Following doublet exclusion, the data was integrated and visualised using UMAP. Clusters were identified based on known markers.

### Bulk RNA sequencing

For bulk RNA sequencing, cultured cells were washed once with PBS, lysed using Trizol and snap-frozen on dry ice. RNA was extracted from the lysates using the TRIzol™ Plus RNA Purification Kit. STAR 2.7.7 was used and reads aligned against UCSC mm10. Count data was fed into deseq2 for differential expression.

### Glucose and Insulin tolerance tests

For glucose tolerance tests, mice were fasted overnight, followed by an intraperitoneal (i.p.) glucose injection at t=0 (1 or 2 g/kg for mice on LFD or HFD respectively). Basal glucose was measured using a hand-held glucometer (Contour next one) via the tail vein prior to the injection, and 15, 30, 45, 60 and 120 minutes following glucose administration. For insulin tolerance tests, mice were fasted for 5 hours in the morning followed by an i.p. injection of insulin (Actrapid) at t=0 (0.5 or 0.75 U/kg for mice on LFD or HFD respectively). Blood measurements were taken as above.

### Body composition measurements

Fat and lean mass of live mice were measured using the echoMRI-100H (EchoMRI).

### Indirect calorimetry

Indirect calorimetry measurements were made using the Oxymax-Comprehensive Lab Animal Monitoring System (CLAMS; Columbus Instruments). Mice were single-housed for two days to acclimatise prior to placing them in the CLAMS cages. Mice were provided with diet and water *ad libitum*. Data was extracted and analysed using the CalR web-based Analysis Tool ( Mina *et al*., 2018), excluding the first 24hr and up to the start of the second night cycle (~29hr) to allow for acclimatisation of the mice. The respiratory exchange ratio (RER) is calculated as ratio between carbon dioxide production (VCO _2_) and oxygen consumption (VO _2_). Energy expenditure (EE) is calculated as follows: *EE* = *VO*2 × *CV*, where *CV* = 3.815 + 1.232 × *RER*. Due to the consistency of the diets used, the mice were able to pull large amounts of food out of the hoppers, resulting in unreliable measures of food consumption and locomotor activity and therefor these results will not be discussed.

### Statistical analysis

Analysis was carried out using GraphPad Prism (GraphPad 9, Dotmatics) unless otherwise specified. Each symbol in scatter dot plots, represents an individual mouse or well depending on the experiment. Bar heights represent means and error bars represent standard deviation (SD). Results where p≤0.05 are considered significant.

## Results

### AHR ligands cause rapid recruitment of AHR to the *Cyp1a1* promoter and AHR pathway activation in mouse adipose tissue *in vivo*

**Figure 1.**
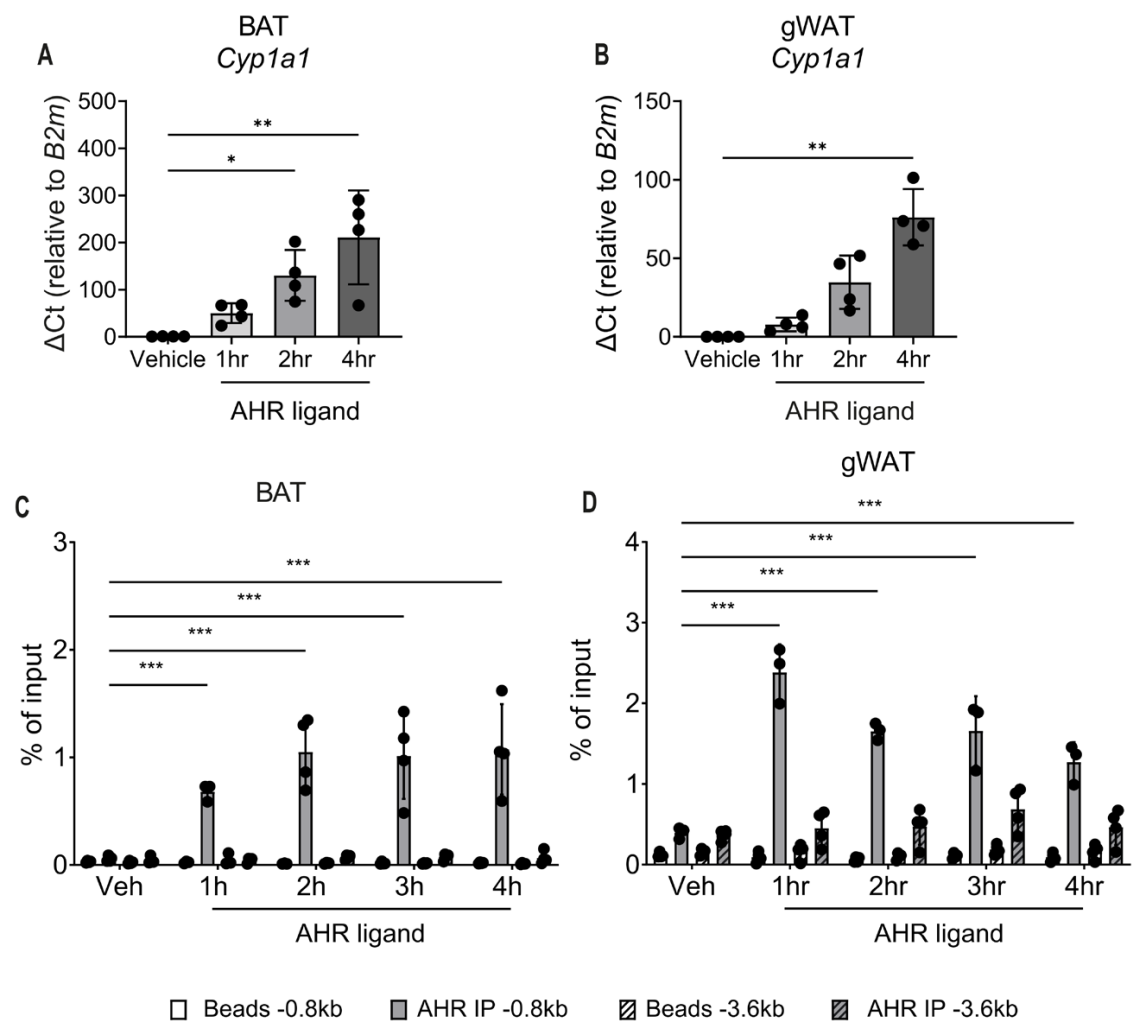
Injected AHR ligands lead to AHR recruitment to the *Cyp1a1* promoter and increased *Cyp1a1* mRNA expression in adipose tissue in mice. **A**– **B**: *Cyp1a1* mRNA expression in brown adipose tissue (BAT; **A**) and perigonadal white adipose tissue (gWAT; **B**) following i.p. injection of vehicle (10% DMSO in corn oil) or 10 mg/kg of the AHR ligand, FICZ, 1, 2 or 4 hours before tissue collection, measured by RT-qPCR. **C**– **D**: ChIP fold enrichment of DNA fragments found at the AHRE region of *Cyp1a1* (-0.8kb) or a negative control region (-3.6kb) in BAT ( **C**) or gWAT ( **D**) from mice treated with vehicle (corn oil) or 26.5 mg/kg of the AHR ligand, 3-MC, i.p. 1– 4 hours before tissue collection, immunoprecipitated using an AHR antibody (AHR IP). Samples were also incubated with beads in the absence of an antibody as another negative control (Beads). Values are expressed as a % of input. **A**– **B**: Data is normalised to B2m expression; Kruskal-Wallis test with Dunn’s multiple comparisons test (n=4). **C**– **D**: Two-way ANOVA with Dunnett’s multiple comparisons test (n=3–4). **A**– **D**: Each symbol represents an individual mouse; bar heights represent means; error bars represent SD; * *P* ≤ 0.05, ** *P* ≤ 0.01, *** *P* ≤ 0.001

First, we aimed to confirm successful AHR ligand delivery to the adipose tissue *in vivo*, through activation of the AHR pathway. Intraperitoneal injection of the AHR ligand 6-formylindolo[3,2-b]carbazole (FICZ), significantly increased the expression of *Cyp1a1*, a direct downstream target of AHR, in mouse brown adipose tissue (BAT) two and four hours after injection, and in perigonadal white adipose tissue (gWAT) four hours after injection ( Figure 1A & B). ChIP-qPCR further demonstrated AHR binding to the *Cyp1a1* promoter region (-0.8kb from the AHRE) in mouse BAT and gWAT within an hour of intraperitoneal injection of the AHR ligand 3-MC ( Figure 1C & D). AHR remained bound to the *Cyp1a1* promoter for at least 4 hours, which was the longest time point measured in this experiment.

### Global AHR KO female mice are protected from diet-induced obesity and the development of metabolic syndrome

Previous studies on a global AHR KO mouse model, have shown that AHR KO mice are protected from DIO and the development of the associated metabolic syndrome ( Wang *et al*., 2011; Xu *et al*., 2015). In agreement, we show that female AHR KO mice gained significantly less weight on a high-fat diet (HFD) than WT mice (Supplemental Figure 1A). This protective phenotype was not observed in male mice (Supplemental Figure 1B). Female AHR KO mice also had a significantly lower fat mass compared to WT mice following 12 weeks on a HFD (Supplemental Figure 1E). There was no significant difference in fat mass in the male AHR KO mice compared to WT (Supplemental Figure 1F). Female AHR KO mice also had improved insulin sensitivity and reduced fasting glucose compared to WT mice following HFD (Supplemental Figure 2B–C). Male AHR KO mice had reduced fasting blood glucose compared to WT mice following a HFD, however, no significant difference in insulin sensitivity was observed (Supplemental Figure 2E–F). No difference in glucose clearance was observed in either male or female AHR KO mice (Supplemental Figure 2A & D). Together this indicates that AHR KO mice may be protected from DIO and metabolic syndrome, in agreement with previous studies.

### Adipocyte-specific AHR deficient mice are not protected from diet-induced obesity or the development of metabolic syndrome

**Figure 2.**
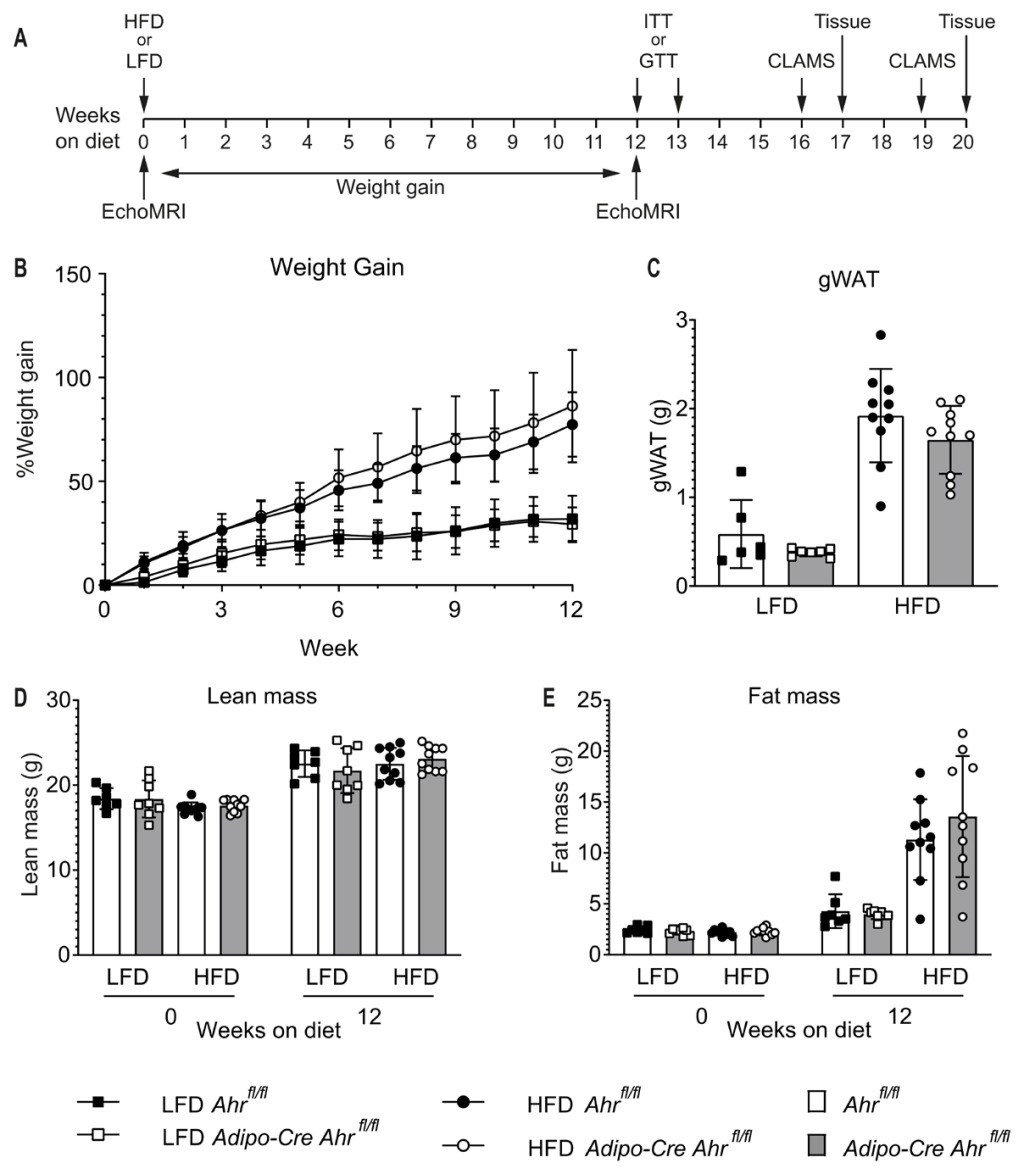
Adipocyte AHR has no effect on diet-induced obesity in the absence of AHR ligands. **A:** Schematic representation of experimental plan. Briefly, male mice were placed on a purified high-fat diet (HFD) or low-fat diet (LFD) at the age of 6–7 weeks and weight was measured weekly for 12 weeks. Body composition was measured by EchoMRI at weeks 0 and 12, and an insulin tolerance test (ITT) and glucose tolerance test (GTT) conducted at weeks 12 and 13. Mice were placed in a Comprehensive Lab Animal Monitoring System (CLAMS) at week 16 or 19 for a week before sacrificing for tissue collection. **B**: Weight-gain of *Adipoq-Cre Ahr ^fl/fl^* male mice and Cre- littermate controls ( *Ahr ^fl/fl^*) fed a HFD or LFD over a period of 12 weeks. **C:** gWAT weight of the aforementioned mice at the time of tissue collection. Lean mass ( **D**) and fat mass ( **E**) of the *Adipoq-Cre Ahr ^fl/fl^* and *Ahr ^fl/fl^* mice at week 0 and 12. **B:** Two-way ANOVA with Turkey’s multiple comparisons test. **C:** Mann-Whitney test **D**– **E:** Two-way ANOVA with Šidák's multiple comparisons test. **C**– **E**: Each symbol represents an individual mouse. Error bars show SD; HFD n=10, LFD n=6–8. The HFD study was repeated twice with comparable results. One repeat is shown in this figure.

Studies have suggested that *Ahr* is expressed in mature adipocytes and that *Ahr* expression in these cells may play a role in DIO and the development of metabolic syndrome ( Baker *et al*., 2015; Haque *et al*., 2023; Huang *et al*., 2022). To test this, we used male *AdipoqCre x AHR ^fl/fl^* mice, in which AHR is deleted in mature adipocytes. The mice were put on a purified low-fat or high-fat diet (LFD, HFD) and weight, body composition and metabolic parameters were measured. As expected, mice placed on a HFD gained weight to a greater degree and had larger gWAT depots and overall increased fat mass when compared to mice placed on a LFD. No effect of genotype was observed in any of those parameters ( Figure 2A–E).

**Figure 3.**
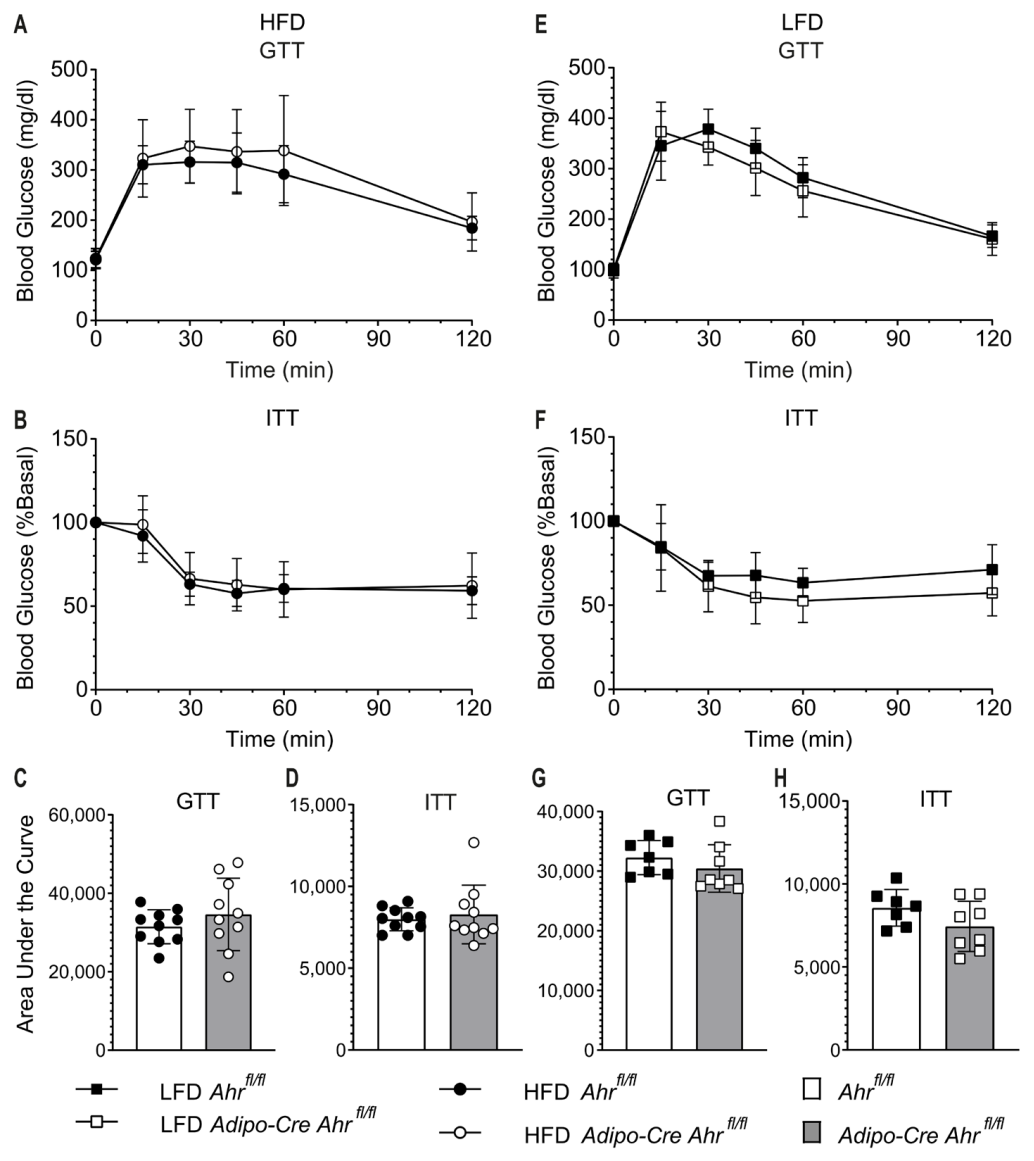
Adipocyte AHR has no effect on the development of metabolic syndrome in the absence of AHR ligands. Glucose tolerance test (GTT) in *Adipoq-Cre Ahr ^fl/fl^* and *Ahr ^fl/fl^* male mice following 12–13 weeks on HFD ( **A**, **C**; 1 g/kg glucose i.p.) or LFD ( **E**, **G**; 2 g/kg glucose i.p.), presented as blood glucose measurements at each timepoint ( **A**, **E**) and area under the curve ( **C**, **G**). Insulin tolerance test (ITT) in *Adipoq-Cre Ahr ^fl/fl^* and *Ahr ^fl/fl^* mice following 12–13 weeks on HFD ( **B**, **D**; 0.75 U/kg insulin i.p.) or LFD ( **F**, **H**; 0.5 U/kg insulin i.p.), presented as % change of blood glucose from basal (t=0) for each timepoint ( **B**, **F**) and area under the curve ( **D**– **H**). A-B, E-F: Two-way ANOVA with Šidák's multiple comparisons test. **C**– **D**, **G**– **H**: Mann-Whitney test; Each symbol represents an individual mouse. Error bars show SD; HFD n=10, LFD n=6–8. The HFD study was repeated twice with comparable results. One repeat is shown in this figure.

**Figure 4.**
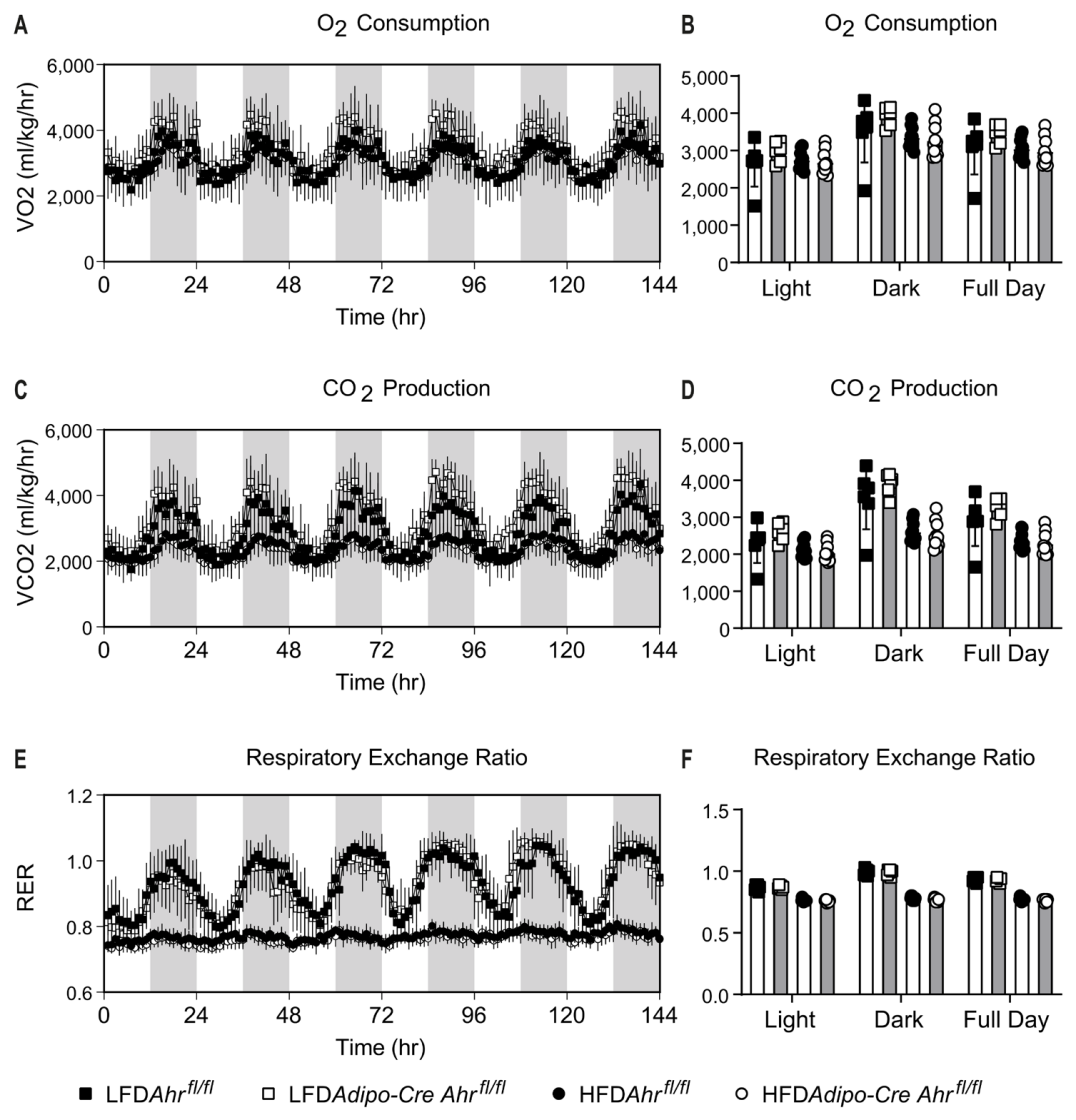
Adipocyte AHR has no effect on whole body metabolism in the absence of AHR ligands. Oxygen consumption ( **A-B**), carbon dioxide production ( **C–D**) and respiratory exchange ratio ( **E–F**) of *Adipoq-Cre Ahr ^fl/fl^* and *Ahr ^fl/fl^* male mice represented as rate for each timepoint ( **A, C, E**) or average rate during the light, or dark cycle, or the whole day ( **B, D, F**), following 13 weeks on HFD. **A, C, E:** White sections represent light and grey sections, dark cycles. **B, D, F:** Two-way ANOVA with Šidák's multiple comparisons test; Error bars show SD; n=10. The HFD study was repeated twice with comparable results. One repeat is shown in this figure.

There were no differences in glucose tolerance or insulin sensitivity ( Figure 3A–H) and no differences in O _2_ consumption, CO _2_ production or respiratory exchange ratio (RER) between genotypes in the LFD or HFD mice ( Figure 4A–F). This experiment was repeated twice with comparable results. As this experiment used purified diets which do not contain significant amounts of AHR ligands, the experiment was repeated using HFD supplemented with the AHR pro-ligand, I3C, in both male and female mice however no significant differences in weight-gain, body composition, glucose tolerance, insulin sensitivity, fasting glucose, O _2_ consumption, CO _2_ production or RER were observed (Supplemental Figure 3–5).

### *Ahr* and *Cyp1a1* expression are undetectable in mature adipocytes

**Figure 5.**
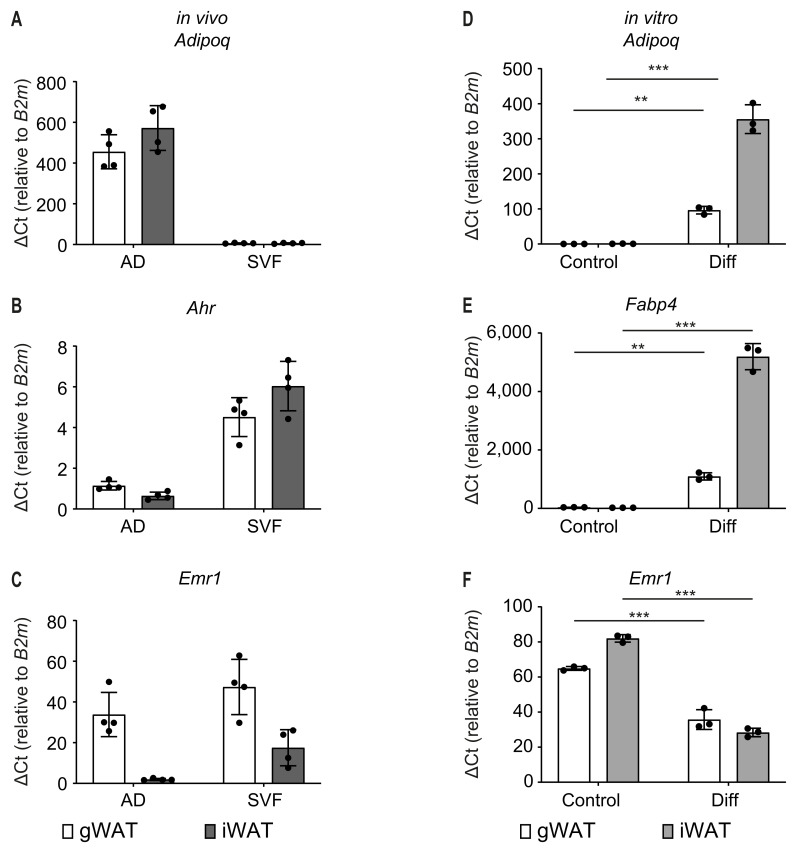
Isolation of adipocytes using the flotation method is not sufficient to remove macrophage contamination. *Adipoq* ( **A**), *Ahr* ( **B**) and *Emr1* ( **C**) mRNA expression in the adipocyte (AD) and SVF fraction separated using the flotation method from gWAT and iWAT of WT male mice. *Adipoq* ( **D**) *, Fabp4* ( **E**) and *Emr1* ( **F**) mRNA expression in SVF progenitor cells isolated from mouse gWAT or iWAT, maintained in normal media (control) or differentiated into mature adipocytes (Diff), measured by RT-qPCR. Data is normalised to B2m expression; Two-way ANOVA with Šidák's multiple comparisons test; each symbol represents an individual mouse; bar heights represent means; error bars represent SD; **A-C**: n=4, **D-F**: n=3; ** p≤0.01, *** p≤0.001.

To further examine *Ahr* expression and activation in mature adipocytes, different techniques were assessed for their ability to isolate mature adipocytes. First, the flotation method was used, which is commonly used to separate adipose tissue into an adipocyte (AD) and a stromovascular fraction (SVF) for downstream analysis. Using qPCR, we show that the AD fraction of both gWAT and inguinal white adipose tissue (iWAT) has a high expression of the mature adipocyte marker, *Adipoq*, which is not present in the SVF ( Figure 5A). *Ahr* expression was higher in the SVF of both tissues, however it was also detectable in the AD fraction ( Figure 5B). Finally, we show that the macrophage marker, *Emr1*, is expressed in both the AD and SVF in gWAT and the SVF of iWAT, and is also low but detectable in the AD fraction of iWAT ( Figure 5C). This demonstrates that the flotation method does not eliminate immune cells from the adipocyte fraction as evidenced by significant macrophage contamination in the AD fraction, especially in gWAT.

Another method for studying mature adipocytes is by differentiating adipocyte progenitors from the SVF cells *in-vitro*. SVF cells isolated using the flotation method from both gWAT and iWAT were successfully differentiated into mature adipocytes in culture, marked by their significant upregulation of *Adipoq* and *Fabp4* ( Figure 5D–E). Despite culture conditions being designed to promote the culture and differentiation of adipocytes, *Emr1* was also highly expressed in both the cells that were kept in maintenance medium and those that underwent the differentiation protocol ( Figure 5F).

**Figure 6.**
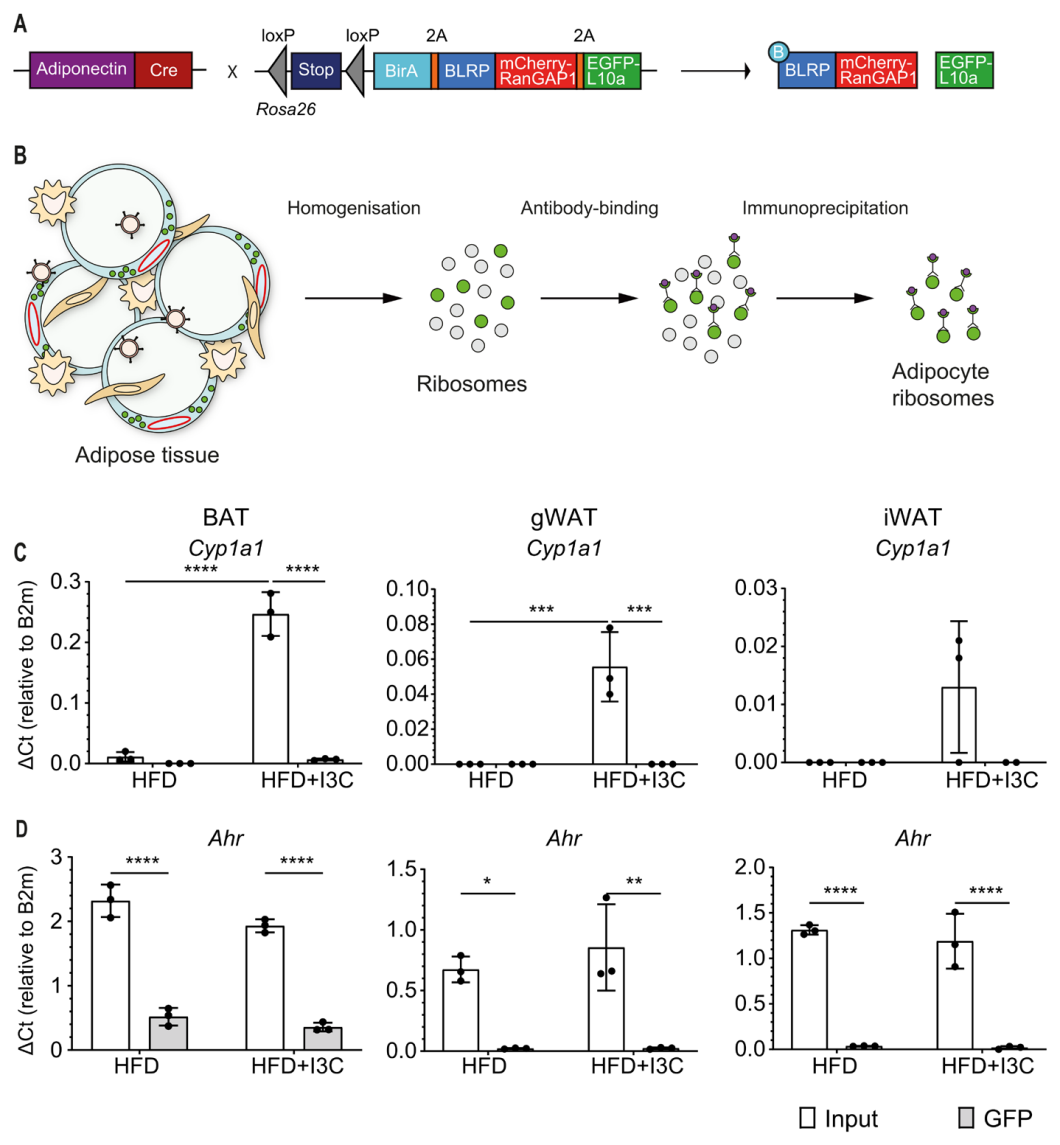
*Ahr* and *Cyp1a1* expression were undetectable in adipocyte ribosomes from mice exposed to AHR ligands. **A**: Schematic representation of the *Adipoq-Cre NuTRAP* mouse. Briefly, this cross results in Cre recombinase cleaving the loxP sites, removing the stop cassette and allowing the expression of the self-cleaving 2A peptides, followed by the biotinylation of BLRP by BirA. This results in EGFP fused to the L10a 60S ribosomal subunit, and mCherry fused to the RanGAP1 nuclear envelope with a biotin tag, expressed exclusively in adipocytes. **B**: Schematic of the TRAP method. Briefly, Homogenised adipose tissue is incubated with anti-GFP antibody-bound dynabeads, which allows for the capture of adipocyte nuclei for downstream analysis. **C**: *Cyp1a1* and *Ahr* ( **D**) RNA expression in adipocyte ribosomes (GFP) and total RNA (input) in BAT, gWAT or iWAT of *Adipoq-Cre NuTRAP* mice fed a purified HFD or HFD supplemented with 1,000 mg/kg I3C for 18 weeks, measured by RT-qPCR (n=3). Two-way ANOVA with Turkey’s multiple comparisons test; Each symbol represents an individual mouse; Error bars show SD.

To more accurately study mature adipocyte gene expression, the *Adipoq-Cre NuTRAP* mouse model was used, in which eGFP is specifically expressed in the ribosomes of mature adipocytes ( Figure 6A). The TRAP method was used to capture mature adipocyte ribosomes, using an anti-GFP antibody, and RT-qPCR used on the input and anti-GFP captured sample to quantify the expression of genes actively translated by mature adipocytes ( Figure 6B). The anti-eGFP-captured mature adipocyte ribosomes showed a high expression of the adipocyte marker, *Adipoq*, and no detectable *Emr1* expression in any of the tissues tested (Supplemental Figure 6A–B). Using this method, we show that, while *Cyp1a1* expression increases in the input sample from adipose tissue of mice injected with the AHR ligand, 3-MC, or fed diet containing the AHR pro-ligand I3C, *Cyp1a1* was not detectable in adipocyte ribosomes of these mice ( Figure 6C, Supplemental Figure 6C). Similarly, while *Ahr* expression is detected in the input sample of these mice, it is not detectable in mature adipocyte ribosomes from gWAT and iWAT tissue ( Figure 6D, Supplemental Figure 6D). Some *Ahr* expression was observed in adipocyte ribosomes from BAT, however *Cyp1a1* expression was barely detectable and did not increase in mice treated with 3-MC or fed I3C ( Figure 6C–D, Supplemental Figure 6C–D). This data suggests that *Ahr* is not expressed in mature white adipocytes and its pathway is not active in mature adipocytes in BAT or WAT.

### Adipose stem and precursor cells make up the majority of Ahr-responsive cells in mouse adipose tissue

**Figure 7.**
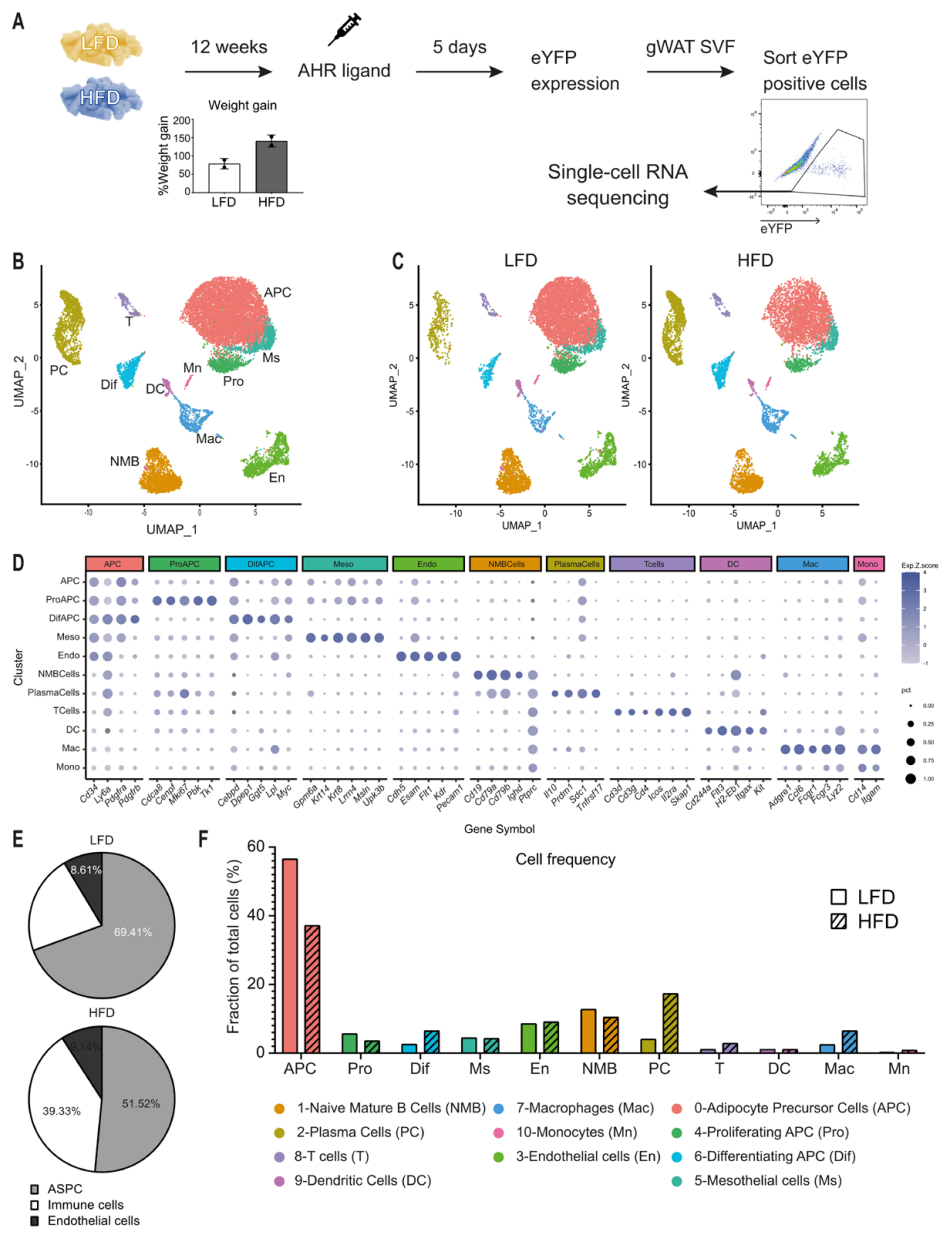
Adipose tissue stem and precursor cells (ASPCs) make up the majority of AHR-ligand-responsive cells in mouse gWAT. **A:** Schematic representation of experimental plan. Briefly, *Cyp1a1* fate reporter male mice were fed a purified LFD or HFD for 12 weeks. These were then injected with FICZ (10 mg/kg) five days prior to gWAT collection. The stromovascular fraction (SVF) was isolated and eYFP+ cells sorted for downstream single-cell RNA sequencing analysis. **B:** UMAP visualisation of the combined data after cut-offs and doublet exclusion applied. **C**: UMAP visualisation of the cell clusters identified in eYFP+ gWAT SVF cells in lean (left) and obese (right). **D:** Dot Plot of the gene expression of selected identifying genes for each cell cluster, where colour intensity represents expression Z score and dot size, percent of cells expressing the selected genes. **E**: Pie chart representing the percentage of adipose stem & precursor cells (ASPC), immune cells and endothelial cells in lean (top) and obese (bottom) mice. **F**: Percentage of cells in each cell cluster identified by scRNAseq in lean (LFD) and obese (HFD) mice. Data from two pooled mice per group.

To further investigate in what cell types in gWAT the AHR pathway is active in, we used *Cyp1a1* fate reporter mice to isolate AHR-responsive (eYFP+) cells from the gWAT SVF of lean and obese mice using Fluorescence-Activated Cell Sorting (FACS; Figure 7A). We then performed single-cell RNA sequencing on these cells to identify AHR-responsive cell types and their transcriptional networks used. We identified three broad cell types that respond to AHR pathway activation with an increase in *Cyp1a1* expression in mouse gWAT: adipose stem and precursor cells (ASPC; clusters 0, 4, 5 and 6), immune cells (clusters 1, 2, 7, 8, 9 and 10) and endothelial cells (cluster 3), defined by their expression of *Pdgfra*, *Ptprc* and *Pecam1* respectively (Supplemental Figure 7A–C). Immune cells identified included B cells, plasma cells, T cells, dendritic cells, macrophages and monocytes. The ASPC clusters included a mesothelial cell cluster, and three adipocyte precursor cell (APC) clusters, including an APC cluster characterized by its expression of proliferation markers such as *Mki67* and a cluster characterized by early differentiation commitment genes, such as *Lpl*, *Cebpd* and *Dpep1* ( Gupta *et al*., 2012; Ntambi & Young-Cheul, 2000) ( Figure 7B–D).

ASPCs made up over half of the cells identified ( Figure 7E), which was confirmed using flow cytometry in an independent experiment (Supplemental Figure 7D–H). In obese mice, an increase in the overall proportion of immune cells, primarily driven by an increase in plasma cells and macrophages, and a decrease in ASPCs, except for the differentiating APC cluster, was observed ( Figure 7C, E–F).

### AHR in adipocyte precursor cells may play a role in adipocyte differentiation

**Figure 8.**
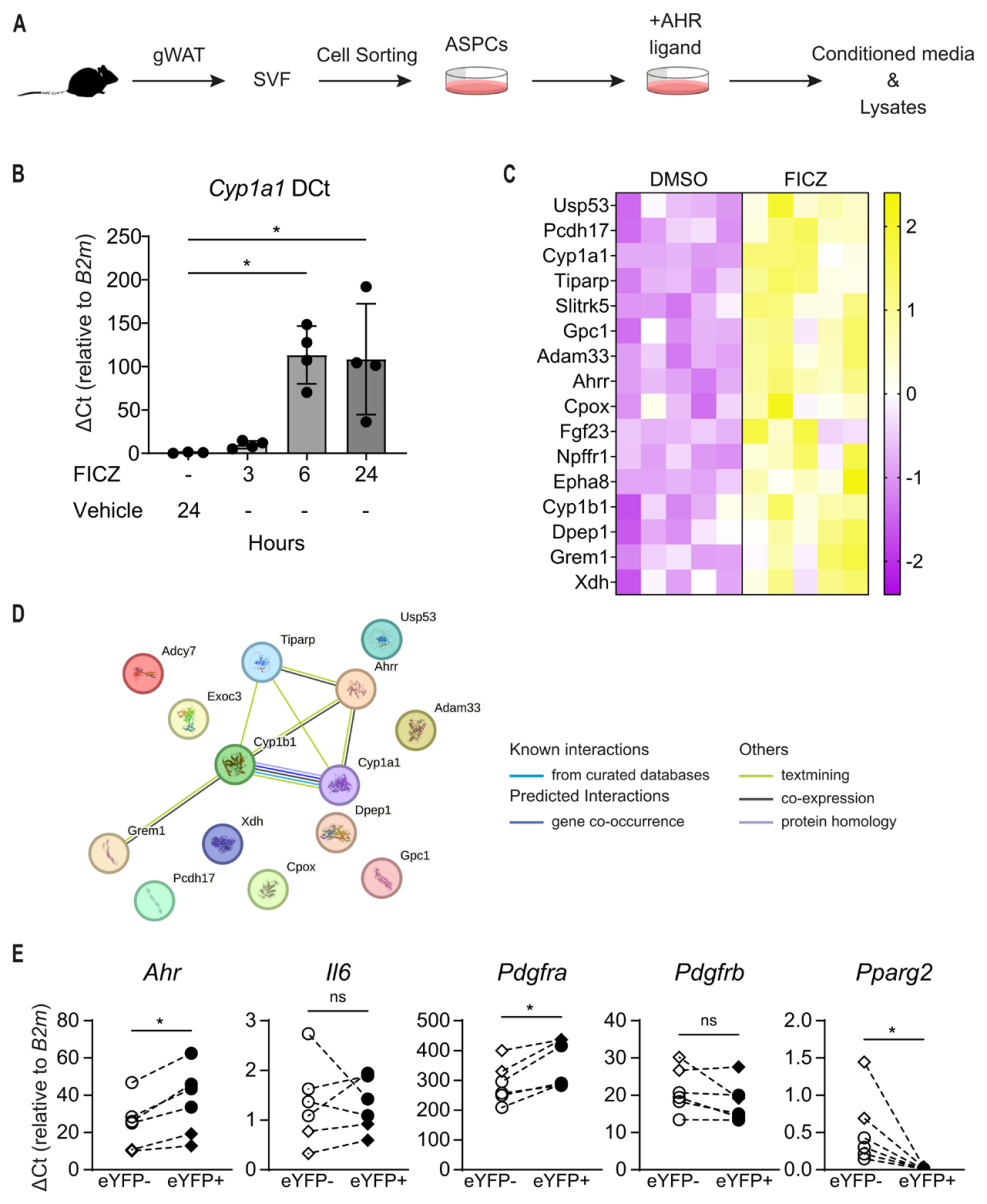
AHR ligands upregulate genes associated with AHR pathway in cultured ASPCs. **A**: Schematic representation of experimental plan. Briefly, gWAT of WT mice was digested and SVF collected. ASPCs were isolated using FACS (CD45-CD31-PDGFRA+) and expanded in culture until confluent. ASPCs were then treated with FICZ (100nM) and the conditioned media and lysates collected 3, 6 or 24hr after treatment for downstream analysis. **B**: *Cyp1a1* mRNA expression in cultured mouse ASPCs following treatment with vehicle or 100nM FICZ as described in **A**, measured by RT-qPCR. Kruskal-Wallis test with Dunn’s multiple comparisons (n=4). Each symbol represents an individual mouse; Error bars show SD. **C**: Heatmap of the RNA expression of the significantly upregulated DE genes in cells treated with DMSO or FICZ for 6hr. Expression is normalized to the average expression of each gene. **D**: Protein-protein interaction map of the DE genes identified by bulk RNAseq, created using STRING (version 12.0). **E**: *Ahr*, *Il6, Pdgfra*, *Pdgfrb* and *Pparg2* mRNA expression in sorted eYFP- and eYFP+ ASPCs from the gWAT of *Cyp1a1* fate reporter mice injected with FICZ (10 mg/kg) five days before tissue collection, measured by RT-qPCR. Wilcoxon matched-pairs signed rank test (n=6). The eYFP- and eYFP+ sample of each mouse is connected by a dashed line.

To further investigate the role of AHR in adipocyte precursors, ASPCs were sorted from the SVF of mouse gWAT and allowed to expand in culture until confluent. Cultured ASPCs were stimulated with the AHR ligand, FICZ, for 3, 6 and 24hr and lysates collected for downstream analysis ( Figure 8A). Using RT-qPCR, we saw a significant upregulation of *Cyp1a1* expression 6hr after stimulation with FICZ, which persisted 24hr after stimulation ( Figure 8B). The 6hr timepoint was selected based on these results for downstream analysis using bulk RNA sequencing. Briefly, ASPCs were sorted as above and expended in cultured. FICZ was added and lysates collected 6hr after stimulation. The significantly upregulated differentially expressed (DE) genes included known downstream targets of AHR, such as *Cyp1a1*, *Cyp1b1*, *Ahrr* and *Tiparp* ( Figure 8C–D). 

To investigate the regulation of a variety of released cytokines and other factors in an unbiased approach, cultured ASPCs were stimulated with the AHR ligand, FICZ, for 24hr and media collected for analysis using the Mouse XL Cytokine Array Kit. The amount of known pro and anti-inflammatory mediators, such as IL-1β, IL-1ra, IL-6, IL-10 and TNFα, appeared to be nearly undetectable, although following quantification, IL-1β and IL-6 appear to be decreased following treatment with FICZ (Supplemental Fig 8A-B). A few other factors (CXCL5, Pentraxin 3, Periostin, Serpin F1, VCAM-1 and VEGEF) were identified that appear to increase following treatment with FICZ (Supplemental Fig 8A–B). Overall, using this assay there were very few factors that appear to change following treatment with FICZ, and as this is a single n, further investigation would be needed to reach a conclusion. Of note, none of the factors identified through the cytokine array were changed at the RNA level in the 6hr bulk RNA sequencing experiment (Supplemental Fig 8C–D).

Finally, we sorted AHR responsive (eYFP+) and non-responsive (eYFP-) ASPCs from *Cyp1a1* fate reporter mice injected with FICZ, using FACS, and saw that ASPCs that have responded to the AHR ligand tend to have a higher expression of *Ahr* and *Pdgfra*, and close to undetectable expression of *Pparg2* ( Figure 8E) which would indicate that *Ahr* expression may play an important part in the early stages of adipocyte differentiation. Overall, this data shows that ASPCs do respond to AHR ligands and provides evidence that warrants further investigation in the function of the AHR pathway in this cell type in the context of inflammation, proliferation and adipocyte differentiation, all of which may play an important role in obesity and the development of metabolic syndrome.

## Discussion

Despite decades of research, obesity continues to rise, with most treatments suffering from lack of adherence, high chances of remission and serious side effects, or the need for surgical intervention ( Look, 2014; van Baak & Mariman, 2019). Over the past few years, the ligand-activated transcription factor, AHR, has received attention as a potential target for the treatment of obesity and metabolic syndrome. Naturally occurring AHR ligands are often derived from diet or produced by gut microbiota, and they are highly lipophilic, resulting in their accumulation in adipose tissue. In this study we show that AHR ligands do indeed rapidly reach adipose depots, leading to AHR pathway activation. As shown by previous studies, we also provide evidence that a global AHR KO mouse model is at least partially protected from DIO and the development of the associated metabolic syndrome. The key finding in our study is that *Ahr* appears to be very lowly or not expressed in mature adipocytes and does not appear to be involved in the development of obesity and metabolic syndrome in these cells. We did observe, however, that ASPCs, including adipocyte precursors, make up the majority of AHR-ligand-responsive cells in adipose tissue which warrants further study into the involvement of AHR in these cells in the context of obesity and metabolism.

Similarly to previous studies, we show that female global AHR KO mice ( Schmidt *et al*., 1996) are protected from DIO and the development of metabolic syndrome (Supplementary Fig 1–2) ( Wang *et al*., 2011; Xu *et al*., 2015). In this study, it is not possible to elucidate whether the improved metabolic phenotype is due to the absence of AHR, or a result of the reduced weight-gain and fat mass in these mice. The only significant differences observed in male AHR KO mice were an increase in lean mass (Supplementary Fig1D) and reduced fasting glucose following 12 weeks on a HFD (Supplementary Fig2F). This is not the first instance of sex differences being observed in the study of AHR in obesity ( Girer *et al*., 2019; Haque *et al*., 2023). AHR has been known to physically interact with the oestrogen receptor α (Erα) ( Beischlag & Perdew, 2005; Gottel *et al*., 2014; Matthews *et al*., 2005; Ohtake *et al*., 2003; Wormke *et al*., 2003), and oestrogen is known to affect obesity and metabolism (Reviewed in Steiner & Berry, 2022). The exact mechanisms behind this interaction and its physiological consequences remain unknown. It is also worth noting that the sample sizes used in conventional mouse pre-clinical studies are limited by practical and financial constraints, which means that small n numbers are the norm. This means studies are not usually powered to detect sex differences and raises the possibility that the sexual dimorphism we and others have observed in AHR KO mice is a false positive result. A study powered to specifically detect sex differences in DIO and metabolism in this model would need to be carried out to investigate whether these reported sex differences are a true effect.

In this study, using a variety of techniques, we show that AHR ligands cause rapid activation of the AHR pathway in spatially and functionally distinct adipose tissue depots, suggesting rapid transportation and uptake of AHR ligands to adipose depots. Using the NuTRAP mouse model, we show that *Ahr* and *Cyp1a1*, a known downstream target of AHR, are not actively translated in mature white adipocytes following *in-vivo* treatment with AHR ligands ( Figure 6 & Supplemental Fig 6). This suggests that *Ahr* is unlikely to be expressed at high enough levels in mature white adipocytes to have a major effect at a whole-tissue or whole-organism level. Low expression of ribosomal *Ahr* expression was observed in mature adipocytes in mouse BAT. Brown adipocytes arise from a distinct type of precursor found in the embryonic mesoderm, expressing *Myf5*, unlike the *Myf5-* white adipocyte precursors ( Seale *et al*., 2008), therefore some differences would be expected between these types of adipocytes. However, despite this observed low ribosomal *Ahr* expression, there was no expression of ribosomal *Cyp1a1* in these cells following treatment with an AHR ligand ( Figure 6C & D & Supplemental Fig 6C & D), and a pilot ChIP study found no AHR binding to the *Cyp1a1* promoter in BAT adipocytes following treatment with an AHR ligand *in-vivo* (unpublished data). It is, therefore, not surprising that we observed no significant differences in the development of obesity or metabolic syndrome in adipocyte specific *Ahr* KO mice fed a HFD with or without the AHR proligand I3C, compared to littermate controls. Further pilot studies in our lab also showed no differences in thermogenesis in female *Adipoq-Cre Ahr ^fl/fl^* mice when presented with an acute cold stimulus or subcutaneous noradrenaline injection compared to WT littermates (Alice Burke, unpublished data). One limitation of our studies we must acknowledge, is that the *Ahr ^fl/fl^* mice we used for the adipocyte-specific *Ahr* deletion experiments and their littermate controls express the low affinity *Ahr* allele, which may make them partly resistant to DIO ( Kerley-Hamilton *et al*., 2012) and may reduce any potential differences between the KO and controls in these experiments. However, despite limitations, taken together, these findings suggest that AHR is not expressed or too lowly expressed in mature adipocytes and therefore is unlikely to play an important role in these cells in the context of obesity, metabolism or thermogenesis.

Another finding from this study we need to highlight, is the lack of specificity of commonly used methods for isolating mature adipocytes. Using the flotation technique, we show that, even though the adipocyte fraction does appear to contain the majority of mature adipocytes and does have some *Ahr* expression, it also contains macrophages. Strikingly, *Emr1* expression in the gWAT adipocyte fraction was comparable to that in the SVF fraction. The gWAT of obese mice contains a high number of crown-like structures composed of macrophages surrounding mature adipocytes, as well as macrophages that have phagocytosed lipids ( Remmerie *et al*., 2020), potentially leading to increased macrophage contamination in the AD fraction of these samples. *Emr1* expression was also observed in adipocytes differentiated *in-vitro* from white adipose tissue SVF. Previous work in our lab (Benjamin Wiggins, unpublished observations), as well as the scRNAseq data we present in this study, have shown that *Ahr* is expressed in white adipose tissue macrophages, therefore we propose that the *Ahr* and *Cyp1a1* expression we observe in these instances may be originating from macrophages and not from mature adipocytes. This means that neither technique is selective enough for mature adipocytes, and therefore it is not possible to confidently conclude that gene expression observed using these techniques originates from mature adipocytes, especially when studying genes highly expressed in macrophages.

Having excluded mature adipocytes as likely to have an effect at the adipose tissue level, we sought to investigate what cells do respond to AHR ligands. We used scRNAseq to identify the AHR-ligand-responsive cells found in the gWAT SVF of lean and obese mice. The *Cyp1a1* reporter mouse model was used to isolate cells in which *Cyp1a1* has been expressed, using FACS. The AHR-responsive cells identified in this experiment included endothelial cells, stomal and precursor cells and a variety of immune cells ( Figure 7). Endothelial cells and certain immune cells have previously been shown to highly express *Ahr*, and the AHR pathway has been shown to be readily activated upon exposure to AHR ligands ( Stockinger *et al*., 2014; Stockinger *et al*., 2021; Wiggins *et al*., 2023), therefore the inclusion of these cells in our scRNAseq results was not surprising. One interesting observation was that the immune cell clusters identified predominantly included cell subtypes associated with anti-inflammatory phenotypes. For example, the plasma cells identified expressed *Ighm* and *Il10*, suggesting they may be a type of regulatory plasma cells, the T cells expressed *Cd4*, *Il13, Gata3* and *Il2ra*, suggesting they may be Th2 cells or Tregs or a mix of both, and the macrophages were characterised by *Arg1* and *Mrc1* expression, suggesting an alternatively activated phenotype. The AHR pathway has been associated with the regulation of anti-inflammatory immune cells, such as *Il10*-expressing B cells ( Piper *et al*., 2019), however, to our knowledge, this has not been investigated in the context of obesity and adipose tissue inflammation and warrants further investigation.

One unexpected finding was that the majority of the AHR-responsive cells in adipose tissue were stromal & precursor cells (ASPC), which made up more than 50% of the AHR-responsive cells in gWAT SVF ( Figure 7). These results were also confirmed at the protein level using flow cytometry (Supplemental Fig 7 D-I). AHR activation by naturally-occurring AHR ligands has been shown to have an anti-inflammatory effect in a variety of cell types ( Lin *et al*., 2019; Piper *et al*., 2019). Using a cytokine array on ASPC conditioned media we show some indications of a potentially anti-inflammatory phenotype in the cells treated with an AHR ligand, however the changes observed were small. Bulk RNAseq of cultured ASPCs showed clear regulation of known AHR downstream targets, such as *Cyp1a1*, *Cyp1b1*, *Tiparp* and *Ahrr*, following stimulation with the AHR ligand, FICZ, demonstrating that these cells are indeed responsive to AHR ligands and suggesting that AHR may play an important role in ASPCs. However, there was no change in pro- or anti-inflammatory cytokine expression in those cells ( Figure 8C & Supplemental Fig 8 D) and no differences were observed in the expression of selected cytokines in sorted AHR-responsive and non-responsive ASPCs ( Figure 8 E), suggesting the predominant function of AHR signaling in these cells may not be related to the regulation of inflammation.

Apart from a higher *Ahr* expression, sorted AHR-responsive ASPCs also had higher *Pdgfra* expression and lower *Pparg2* expression compared to the non-AHR-responsive ASPCs ( Figure 8 E). Interestingly, both genes have been suggested to be involved in the regulation of differentiation in adipocyte precursors. Studies have suggested that the balance between *Pdgfrb* and *Pdgfra* expression in adipocyte precursors may play an important role in their ability to change from a proliferative, stem-like, phenotype to an adipogenic phenotype. Gao *et al* suggest that *Pdgfra* expression precedes *Pdgfrb* expression and that white adipocytes following HFD develop from *Pdgfrb+* ASPCs, while Sun *et al* demonstrate that, although *Pdgfra* is expressed uniformly in APCs, *Pdgfrb* is not expressed in the more stem-like APCs ( Ferrero *et al*., 2020; Gao *et al*., 2018; Sun *et al*., 2020). Indeed, in our sc-RNAseq data, the cluster with the highest *Pdgfrb* expression was the one that was found to express the early differentiation genes ( Figure 7; DifAPC). *Pparg2* is often referred to as the master regulator of adipogenesis ( Zhang *et al*., 2004). This means that its lack of expression in AHR-responsive ASPCs in our study may support the hypothesis that AHR-responsive ASPCs may be of a less adipogenic nature. In support of that, Dou *et al* suggest that AHR may regulate adipogenesis through the regulation of PPARγ stability ( Dou *et al*., 2019). Several studies have suggested AHR may play an important part in adipocyte differentiation, showing an inhibition of differentiation by addition of AHR ligands such as TCDD or DIM, or genetic overexpression of AHR in the adipocyte precursor cell line, 3T3-L1 in-vitro, while knocking out *Ahr* was shown to increase adipogenesis ( Chen *et al*., 1997; Dou *et al*., 2019; Phillips *et al*., 1995; Shimba *et al*., 1998; Yang *et al*., 2017). Studies have also demonstrated that *Ahr* expression in 3T3-L1 cells falls following induction of adipogenesis and remains low in mature adipocytes, while the expression of the negative regulator of AHR signalling, *Ahrr* increases ( Alexander *et al*., 1998; Brodie *et al*., 1996; Chen *et al*., 1997; Dou *et al*., 2019; Ishihara *et al*., 2018; Phillips *et al*., 1995; Shimba *et al*., 1998). Taken together, this suggests that AHR in ASPCs may play an important role in the regulation of adipogenesis, which could play a key role in the development of metabolically healthy adipose tissue.

In conclusion, results from our study suggest that *Ahr* expression in adipose tissue, apart from immune and endothelial cells, is more likely derived from adipocyte precursors rather than mature adipocytes. In the latter, it appears largely absent, and deletion of *Ahr* in mature adipocytes appears to have no functional consequences. We therefore propose that future studies designed to identify AHR-related treatments for obesity focus on the role of AHR in adipocyte precursor cells and adipogenesis.

## Data Availability

Sequencing data generated as part of this manuscript have been deposited on GEO as a SuperSeries (accession number GSE311599), containing SubSeries under accession numbers GSE311153 and GSE311598. Gene Expression Omnibus: Activation of the aryl hydrocarbon receptor in mature adipocytes does not impact diet-induced obesity in mice. Link: https://www.ncbi.nlm.nih.gov/geo/query/acc.cgi?acc=GSE311599 ( Bioinformatics Core, 2025) GSE311153: Bulk RNA sequencing data from cultured mouse adipocyte precursor cells GSE311598: Single-cell RNA sequencing data from FACS isolated mouse adipose tissue stromovascular eYFP+ cells The supplemental figures, html of the sequencing data and the ARRIVE checklist were deposited on OSF as described below. Open Science Framework: Activation of the aryl hydrocarbon receptor in mature adipocytes does not impact diet-induced obesity in mice. Link: https://osf.io/dx3fj/overview ( Vlachaki Walker, 2025) This project contains the following underlying data: Supplemental Figures.pdf: Supplemental Figures 1–8 BulkRNAseq_FICZS_vs_DMSO.html: QC and processing of the bulk RNA sequencing data used in this paper scRNAseq_WAT_FocusRes0.3.html: QC and processing of the single-cell RNA sequencing data used in this paper Author Checklist - Full-JVW.pdf: ARRIVE 2.0 checklist The data is available under the terms of the Creative Commons Zero "No rights reserved" data waiver (CC0 1.0 Public domain dedication).
