# Supplementary material for: Mature adipocytes lack functional Aryl Hydrocarbon Receptor – a study investigating its role in diet-induced obesity in mice
Source: Wellcome Open Res. 2026 Jun 24;11:114. Originally published 2026 Feb 16. [Version 2] doi: 10.12688/wellcomeopenres.25201.2 (PMC13310359; doi:10.12688/wellcomeopenres.25201.2)
Supplement: Peer review history

# Reviewer report: Reviewer response for version 2

**DOI:** 10.21956/wellcomeopenres.29505.r159044

**Published:** 2026-06-27

**Authors:** Josephine Robb

**Affiliations:** 1 University of Exeter, Exeter, England, UK

The authors have addressed all the concerns I raised.

Is the work clearly and accurately presented and does it cite the current literature?

Yes

If applicable, is the statistical analysis and its interpretation appropriate?

Yes

Are all the source data underlying the results available to ensure full reproducibility?

Yes

Is the study design appropriate and is the work technically sound?

Partly

Are the conclusions drawn adequately supported by the results?

Yes

Are sufficient details of methods and analysis provided to allow replication by others?

Yes

Reviewer Expertise:

DIO and metabolic syndrome in mouse genetic models

I confirm that I have read this submission and believe that I have an appropriate level of expertise to confirm that it is of an acceptable scientific standard.

# Reviewer report: Reviewer response for version 2

**DOI:** 10.21956/wellcomeopenres.29505.r159043

**Published:** 2026-06-27

**Authors:** Emiel van der Vorst

**Affiliations:** 1 RWTH Aachen University, Aachen, Germany

The authors have addressed my concerns appropriately. Although not everything could be addressed experimentally, limitations have at least been clearly stated.

Is the work clearly and accurately presented and does it cite the current literature?

Yes

If applicable, is the statistical analysis and its interpretation appropriate?

Yes

Are all the source data underlying the results available to ensure full reproducibility?

Partly

Is the study design appropriate and is the work technically sound?

Yes

Are the conclusions drawn adequately supported by the results?

Partly

Are sufficient details of methods and analysis provided to allow replication by others?

Yes

Reviewer Expertise:

Cardiovascular disease; Atherosclerosis; Aryl Hydrocarbon Receptor

I confirm that I have read this submission and believe that I have an appropriate level of expertise to confirm that it is of an acceptable scientific standard.

# Reviewer report: Reviewer response for version 1

**DOI:** 10.21956/wellcomeopenres.27777.r152009

**Published:** 2026-04-23

**Authors:** Emiel van der Vorst

**Affiliations:** 1 RWTH Aachen University, Aachen, Germany

The manuscript by Walker et al. investigate the role of AhR in mature adipocytes in the context of diet-induced obesity and observe that the AhR in mature adipocytes does not play a role. They highlight that AhR is rather expressed in progenitor cells and not in mature cells, which most likely underlies the observed lack of the effects.

 The study is very well conducted and clearly described. 

 Some critical points that should be taken into account are the following:

- Throughout the manuscript, different AhR ligands are used for example in Figure 1 for the Cyp1 expression and the ChIP analysis, but also for the in-vivo experiments in Figure 6 and 7. Ideally, the full analysis is shown for both ligands to avoid ligand-bias. Alternatively, the figures should reflect this even better, and notions have to be included in the text to highlight the changed ligands.
- Sex effects are not investigated throughout the manuscript with selective use of sexes, which is a shame, as in the global KO, there seem to be clear sex-dependent effects.
- Figure 5 should be shifted to the supplement as it is for protocol optimization, rather than providing outcomes.

Is the work clearly and accurately presented and does it cite the current literature?

Yes

If applicable, is the statistical analysis and its interpretation appropriate?

Yes

Are all the source data underlying the results available to ensure full reproducibility?

Partly

Is the study design appropriate and is the work technically sound?

Yes

Are the conclusions drawn adequately supported by the results?

Partly

Are sufficient details of methods and analysis provided to allow replication by others?

Yes

Reviewer Expertise:

Cardiovascular disease; Atherosclerosis; Aryl Hydrocarbon Receptor

I confirm that I have read this submission and believe that I have an appropriate level of expertise to confirm that it is of an acceptable scientific standard, however I have significant reservations, as outlined above.

# Response

**Published:** 2026-06-05

**Authors:** Julia Vlachaki Walker

**Affiliations:** King's College London Institute of Psychiatry Psychology & Neuroscience, London, England, UK

We thank the reviewer for taking the time to review our manuscript and raising important points, especially regarding the AHR ligands used. Please see the point-by point responses to the reviewer's comments and suggestions:

**Some critical points that should be taken into account are the following:**

 Throughout the manuscript, different AhR ligands are used for example in Figure 1 for the Cyp1 expression and the ChIP analysis, but also for the in-vivo experiments in Figure 6 and 7. Ideally, the full analysis is shown for both ligands to avoid ligand-bias. Alternatively, the figures should reflect this even better, and notions have to be included in the text to highlight the changed ligands.

- The ligand names have been added to figures 1, 7 and 8. A section has been added to the discussion to highlight the potential differences between these ligands.

Sex effects are not investigated throughout the manuscript with selective use of sexes, which is a shame, as in the global KO, there seem to be clear sex-dependent effects.

- We agree with the reviewer that these findings would be much stronger if all diet experiments had been done consistently in both sexes. Sadly, not enough mice were available at the time.

Figure 5 should be shifted to the supplement as it is for protocol optimization, rather than providing outcomes.

- While figure 5 was used for protocol optimisation, it contains some key data demonstrating that techniques commonly used to isolate mature adipocytes may be introducing significant macrophage contamination. We believe this is an important finding for the field, which deserves attention. Moreover, it is also this finding which then led us to use more specific isolation techniques like TRAP. Overall, therefore, if this reviewer is not strongly opposed, we would prefer to keep the Figure within the main manuscript text.

# Reviewer report: Reviewer response for version 1

**DOI:** 10.21956/wellcomeopenres.27777.r152015

**Published:** 2026-04-22

**Authors:** Josephine Robb

**Affiliations:** 1 University of Exeter, Exeter, England, UK

This study investigates the whether the effects of loss of the transcription factor aryl hydrocarbon receptor (AHR) on obesity and metabolic syndrome are mediated through mature adipocytes. This adds to and builds upon previous studies, demonstrating that global loss of AHR decreases fasting glucose levels and decreases fat mass in females. To investigate whether this is mediated by activation of AHR in mature adipocytes, they utilise a AdipoqCre Ahr fl/fl mouse model, to specifically remove Ahr expression from mature adipocytes. They do not observe any effect on body weight or metabolism as a consequence of this loss. They then go on to demonstrate that Ahr is not expressed in mature adipocytes, and that activation of AHR can trigger differentiation of adipocyte precursor cells. 

 I have a concern with the animal studies presented. As the studies in Figure 2-4 were conducted in males, and no phenotype (weight gain or glucose clearance) had been observed in the global knockout in males (supplementary figures 1 & 2) this may suggest that the lack of effect reported is due to the use of males and an effect may have been observed in females. Whilst female fl/fl animals were included in supplementary fig 3 & 4, no HFD controls without I3C were included. This makes it difficult to interpret this dataset as both loss of Ahr and I3C treatment are expected to decrease weight gain, which may be the reason for the lack of phenotype. However, it is unlikely for females to present a phenotype if also females lack AHR in mature adipocytes. Therefore, can the authors clarify whether the assessment of Ahr expression in Figure 6 was conducted on male or female mice, or both? If this was only conducted in males, and as the scRNAseq data presented in Fig. 7 is from males only, is it possible for the authors to validate whether or not Ahr expression is present in female mature adipocytes?

 Aside from this, I believe the data demonstrating that Ahr is not expressed or functional in mature adipocytes are convincing and the finding are important for the field.

 I believe a few minor changes may increase the clarity of this manuscript:

 In Figure 1 it is not immediately obvious that two separate ligands were used for these experiments. Labels on the figure should be changed to indicate which ligand was used in which experiment. The time the animals were treated with vehicle is not clear and should be indicated to allow determination of whether it is an appropriate control. The sex of the animals used should be indicated as there is a potential sex difference in phenotype reported with the Ahr global knock out.

 It is unclear whether the effect of diet on % weight gain, gWAT and fat mass after 12 weeks on diet (Fig 2B, C, E) was not statistically significant or if it has just been omitted from the graph, so clarification in the figure or in the text would be appropriate.

 In Figure 5D it is unclear which comparison the upper asterixis are indicating. I assume that it is the comparison between iWAT groups, but please adjust as appropriate.

 I would also recommend amending the title to more accurately reflect the main finding, that mature adipocytes do not express functional Ahr.

Is the work clearly and accurately presented and does it cite the current literature?

Yes

If applicable, is the statistical analysis and its interpretation appropriate?

Yes

Are all the source data underlying the results available to ensure full reproducibility?

Yes

Is the study design appropriate and is the work technically sound?

Partly

Are the conclusions drawn adequately supported by the results?

Yes

Are sufficient details of methods and analysis provided to allow replication by others?

Yes

Reviewer Expertise:

DIO and metabolic syndrome in mouse genetic models

I confirm that I have read this submission and believe that I have an appropriate level of expertise to confirm that it is of an acceptable scientific standard, however I have significant reservations, as outlined above.

# Response

**Published:** 2026-06-05

**Authors:** Julia Vlachaki Walker

**Affiliations:** King's College London Institute of Psychiatry Psychology & Neuroscience, London, England, UK

We thank the reviewer for taking the time to review our manuscript and raising important points, especially regarding the sex of the mice. Please see the point-by point responses to the reviewer's comments and suggestions:

 I have a concern with the animal studies presented. As the studies in Figure 2-4 were conducted in males, and no phenotype (weight gain or glucose clearance) had been observed in the global knockout in males (supplementary figures 1 & 2) this may suggest that the lack of effect reported is due to the use of males and an effect may have been observed in females. Whilst female fl/fl animals were included in supplementary fig 3 & 4, no HFD controls without I3C were included. This makes it difficult to interpret this dataset as both loss of Ahr and I3C treatment are expected to decrease weight gain, which may be the reason for the lack of phenotype. However, it is unlikely for females to present a phenotype if also females lack AHR in mature adipocytes. Therefore, can the authors clarify whether the assessment of Ahr expression in Figure 6 was conducted on male or female mice, or both? If this was only conducted in males, and as the scRNAseq data presented in Fig. 7 is from males only, is it possible for the authors to validate whether or not Ahr expression is present in female mature adipocytes?

- We thank the reviewer for highlighting this. We did not communicate clearly enough that we do have these crucial results for both sexes, i.e. figure 6 data are from both females (main manuscript) and males (supplemental figure 6). Both figure legends have been updated to include the sex of the mice used in these experiments.

Aside from this, I believe the data demonstrating that Ahr is not expressed or functional in mature adipocytes are convincing and the finding are important for the field.

 I believe a few minor changes may increase the clarity of this manuscript:

 In Figure 1 it is not immediately obvious that two separate ligands were used for these experiments. Labels on the figure should be changed to indicate which ligand was used in which experiment. The time the animals were treated with vehicle is not clear and should be indicated to allow determination of whether it is an appropriate control. The sex of the animals used should be indicated as there is a potential sex difference in phenotype reported with the Ahr global knock out.

- The ligand names have been added to the graphs, and the vehicle treatment time has been added to the figure legend. Unfortunately, the sex of the mice was not recorded for this experiment.

It is unclear whether the effect of diet on % weight gain, gWAT and fat mass after 12 weeks on diet (Fig 2B, C, E) was not statistically significant or if it has just been omitted from the graph, so clarification in the figure or in the text would be appropriate.

- The statistics have been updated to include the comparison between diet groups, and the annotation on the graph and statistics on the figure legend have been updated to reflect this.

In Figure 5D it is unclear which comparison the upper asterixis are indicating. I assume that it is the comparison between iWAT groups, but please adjust as appropriate.

- We thank the reviewer for noticing and highlighting this mistake. The annotation has been adjusted.

I would also recommend amending the title to more accurately reflect the main finding, that mature adipocytes do not express functional Ahr.

- We have amended the title to: “Mature adipocytes lack functional Aryl Hydrocarbon Receptor – a study investigating its role in diet-induced obesity in mice”
